# Supplementary material for: Simulation analysis of fertilizer discharge process using the Discrete Element Method (DEM)
Source: PLoS One. 2020 Jul 16;15(7):e0235872. doi: 10.1371/journal.pone.0235872 (PMC7365441; doi:10.1371/journal.pone.0235872)
Supplement: S1 File — (PDF) [file pone.0235872.s001.pdf]

Supporting information

S1 file

Simulated discharge mass rate (g/s)

Simulation run	Spiral grooved-wheel		Straight grooved-wheel	
	Compound fertilizer	Urea fertilizer	Compound fertilizer	Urea fertilizer
1	105.73	70.19	105.52	89.1
2	108.5	88.41	98.21	101.97
3	117.02	103.95	93.36	59.5
4	115.99	61.58	88.74	72.07
5	120.38	57.22	90.88	60.49

Test discharge mass rate (g/s)

Test run	Compound fertilizer		Urea fertilizer	
	Spiral wheel	Straight wheel	Spiral wheel	Straight wheel
1	632.46	534.6	582.46	534.6
2	589.26	481.82	563.26	481.82
3	600.16	357	577.16	363
4	558.44	432.42	558.44	432.42
5	545.28	362.94	545.28	362.94

Simulated fertilizer falling velocity of two groove wheels (m/s)

Time (s)	Falling velocity for compound fertilizer		Falling velocity for urea fertilizer	
	Spiral wheel	Spiral wheel	Spiral wheel	Straight wheel
0	0	0	0	0
0.1	1.21	1.01	1.2	1.09
0.3	1.1	0.8	1.19	0.88
0.4	0.8	1.2	1	1.08
0.5	1.3	1.21	1.29	1.19
0.7	1	0.9	1.09	0.98
0.8	0.9	1.13	0.99	1.21
0.9	1.2	1.1	1.29	1.18
1.1	0.9	0.89	0.99	0.97
1.2	1.2	0.91	1.29	0.99
1.3	1.11	1.2	1.2	1.28
1.5	1.21	0.8	1.3	1
1.6	1.14	1	1.23	1.08

1.7	1	1.11	1.09	1.19
1.9	1.18	0.91	1.27	0.99
2	1	0.85	1.09	0.93
2.1	0.99	0.88	1.08	0.96
2.3	1.11	0.99	1.2	1.07
2.4	1.15	1	1.24	1.08
2.5	0.98	1	1.07	1.08
2.7	1.17	1.09	1.26	1.17
2.8	1	0.92	1.09	1
2.9	0.98	1.1	1.07	1.18
3.1	0.95	1.16	1.04	1.24
3.2	1.2	0.99	1.29	1.07
3.3	0.98	0.96	1.07	1.04
3.4	1.22	1.1	1.31	1.18
3.6	1.2	1	1.29	1.08
3.7	1.09	0.99	1.18	1.07
3.8	1.07	0.9	1.16	0.98
4	1.16	0.98	1.25	1.06
4.1	0.99	1.1	1.08	1.18
4.2	1.2	1	1.21	1.08
4.4	1.2	0.93	1.29	1.01
4.5	1	0.97	1.09	1.05
4.6	1.09	0.99	1.18	1.07
4.8	1.4	0.95	1.3	1.03
4.9	1.25	0.91	1.34	0.96
5	1	0.88	1.09	0.99
5.2	1.15	0.98	1.24	1.04
5.3	1.11	0.97	1.2	1.07
5.4	1	0.96	1.09	1.03
5.6	0.89	0.95	0.98	1.04
5.7	0.9	0.88	0.99	1.06
5.8	1.05	0.98	1.14	1.2
6	1	0.9	1.09	1.14
6.1	1.1	0.94	1.19	0.98
6.2	1	0.95	1.09	1.08
6.4	1	1	1.09	1.13
6.5	1.1	0.99	1.19	1.07

Measured fertilizer falling velocity of two groove wheels (m/s)

Time (s)	Falling velocity for compound fertilizer		Falling velocity for urea fertilizer	
	Spiral wheel	Spiral wheel	Spiral wheel	Straight wheel
0	0	0	0	0
1.2	1.3	0.74	0.94	0.796
2.4	1.2	0.68	1.2	0.736
3.6	0.98	0.64	0.98	0.9
4.8	1.4	0.89	1.09	0.946
6	1.106	0.8	1.17	1.026
7.2	1.2	0.76	1.3	0.816
8.4	1.4	0.83	1.03	0.886
9.6	1.2	0.69	1.2	0.95
10.8	1.076	0.82	1.14	0.996
12	1.25	0.88	1.08	0.936
13.2	1.08	0.78	1.256	1
14.4	1.02	0.996	1.196	0.94
15.6	1.4	0.956	1.156	0.9
16.8	1.2	0.746	0.946	0.81
18	1.2	0.986	1.186	0.93
19.2	1.07	1.006	1.106	0.95
20.4	1.08	1.056	1.156	1
21.6	1.3	0.926	1.026	0.91
22.8	1.3	1.016	1.116	1
24	1.09	0.846	0.946	0.83
25.2	0.94	0.916	1.016	0.9
26.4	1.13	0.916	1.04	0.84
27.6	1.12	0.916	1.016	1.08
28.8	1.13	0.796	1.2	1.09
30	1.02	0.736	1.2	0.98
31.2	0.92	0.696	1.15	0.88
32.4	1.22	0.946	1.3	0.8
33.6	0.92	1.026	1.126	0.88
34.8	1.1	0.816	0.916	0.78
36	1.12	0.886	0.986	1.08
37.2	1.18	0.746	1.046	0.78
38.4	1.12	0.996	1.096	1.08
39.6	1.03	0.936	1.036	0.99
40.8	1.13	1.056	1.156	0.79
42	1.3	0.996	1.096	1.02
43.2	1.2	0.956	1.056	0.88
44.4	1.1	0.746	1.2	1.06
45.6	1.2	0.986	1.086	0.96
46.8	1.1	1.006	1.106	0.95
48	1.03	1.056	1.156	1.07
49.2	1.07	0.926	1.026	0.78
50.4	1.04	0.98	1.116	0.94

51.6	1.09	0.846	1.13	0.98
52.8	1.3	1.026	1.126	0.96
54	1.34	0.816	0.916	0.94
55.2	1.38	0.886	0.986	0.81
56.4	1.12	0.746	1.12	1
57.6	1.3	1.12	1.28	1.18
58.8	1.08	0.996	1.096	0.94
60	1.14	0.936	1.036	0.92

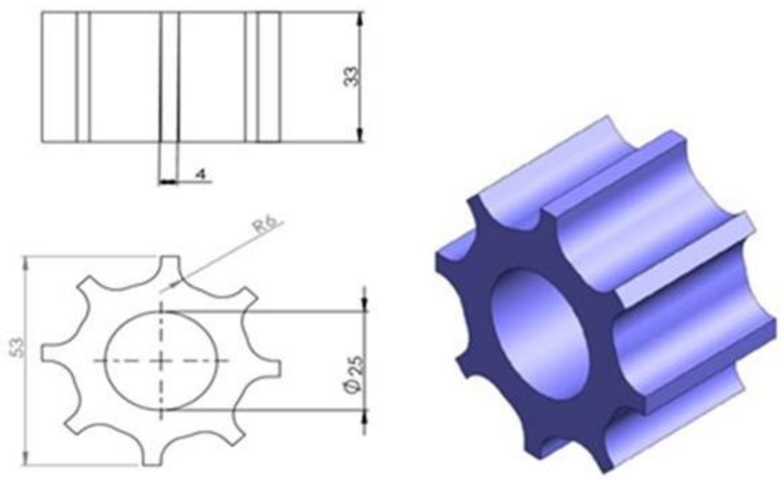

Main structural dimensions and model of the straight grooved-wheel
