# Supplementary material for: Simulation analysis of fertilizer discharge process using the Discrete Element Method (DEM)
Source: PLoS One. 2020 Jul 16;15(7):e0235872. doi: 10.1371/journal.pone.0235872 (PMC7365441; doi:10.1371/journal.pone.0235872)
Supplement: Attachment — Submitted filename: Comments.docx [file pone.0235872.s002.docx]

The manuscript’s topic deals with the testing and simulation of fertilizer discharge process of a newly developed discharge device versus a conventional one. The topic of the manuscript falls within the general scope of the journal and contains potentially useful information for readers. The use of the simulation method of discrete element method is innovative. Generally speaking, the manuscript is acceptable for publication after some minor revisions.

1. Line 79, spell out ‘Fig’ at the beginning of the sentence
2. Tables, please remove all vertical lines and some of unnecessary horizontal lines, refer to the journal guidance
3. Tables 2 and 3, please specify the sources of each DEM parameter specifically in the tables
4. Line 140, ‘given the running time of 60 seconds’ redundant information, suggest deleting
5. Line 156, please provide the brand and model of the camera used
6. Line 160, please explain what is ‘application 3.1’
7. How to calculate the theoretical fertilizer discharge?
8. In actual operation, the fertilizer distributor has a forward movement speed. How does it affect the parameter information simulated in the article?
9. What is the basis for selecting the height parameter of equipment setting?
