# Supplementary material for: Simulation analysis of fertilizer discharge process using the Discrete Element Method (DEM)
Source: PLoS One. 2020 Jul 16;15(7):e0235872. doi: 10.1371/journal.pone.0235872 (PMC7365441; doi:10.1371/journal.pone.0235872)
Supplement: Attachment — Submitted filename: Response to Reviewers comments.docx [file pone.0235872.s003.docx]

**Response to Reviewer**

We appreciate the reviewer’s comments. The followings are our point-by-point responses:

1. Line 79, spell out ‘Fig’ at the beginning of the sentence

Response: Fig has been spelt out in line 82

2. Tables, please remove all vertical lines and some of unnecessary horizontal lines, refer to the journal guidance

Response: We corrected the tables as indicated

3. Tables 2 and 3, please specify the sources of each DEM parameter specifically in the tables

Response: We have specified the source of each DEM parameters in tables 2 and 3.

4. Line 140, ‘given the running time of 60 seconds’ redundant information, suggest deleting

Response: The sentence ‘given the running time of 60 seconds’ has been deleted in line 143.

5. Line 156, please provide the brand and model of the camera used

Response: We have provided the brand and model of the camera used in line 159.

6. Line 160, please explain what is ‘application 3.1’

Response: ‘application 3.1’ has been corrected to application software version 3.1 in line 164.

7. How to calculate the theoretical fertilizer discharge?

Response: We have added information to the paper (page 10, lines 181 – 190) to explain how to calculate the theoretical fertilizer discharge.

8. In actual operation, the fertilizer distributor has a forward movement speed. How does it affect the parameter information simulated in the article?

Response: We agree with the reviewer that in actual operation, the fertilizer distributor has a forward movement speed. However, the article was tailored to compare the discharge performance of the conventional straight grooved-wheel and the spiral grooved-wheel through simulations and platform tests. The platform test and simulation results indicated the validity and effectiveness of DEM analysis on the operation of the spiral grooved-wheel fertilization device as demonstrated in the article. Our next research plan as mentioned in the conclusions section of this article (lines 343 – 345) will focus on adopting the developed spiral grooved-wheel fertilizer distributor on a two-row rice transplanter, considering the forward movement speed of the transplanter. The effect of forward movement speed on discharge performance and accurate fertilizer application rates will be assessed. This will further prove the effectiveness of the simulation model in the article.

9. What is the basis for selecting the height parameter of equipment setting?

Response: As requested by the reviewer, we have added four sentences to the paper (lines 73 – 76) to explain the basis for selecting the height of the equipment setting.
